# Lichen sclerosus of the eyelid involving the eyelash margin

**DOI:** 10.1002/ski2.368

**Published:** 2024-03-16

**Authors:** Tiffany Ho, Susana Ortiz Urda, Ryan S. Berry

**Affiliations:** ^1^ Department of General and Surgical Dermatology Santa Barbara Skin Institute Santa Barbara California USA; ^2^ Department of Dermatology University of California San Francisco San Francisco California USA; ^3^ Spartia Therapeutics Inc. San Francisco California USA; ^4^ Western Pathology Inc. San Luis Obispo California USA

## Abstract

Lichen sclerosus is a debilitating and chronic disease that typically affects the anogenital area, although it can also be found on extragenital locations such as the shoulders, neck, trunk, breasts, and arms. Facial involvement is rare, but there have been a few reported cases of extragenital lichen sclerosus affecting the infraorbital area. To our knowledge, there are 7 documented cases of extragenital lichen sclerosus affecting the eyelid in medical literature. This is a novel case and documented report of a patient with extragenital lichen sclerosus located on the eyelid with eyelash margin involvement.

## Graphical abstract

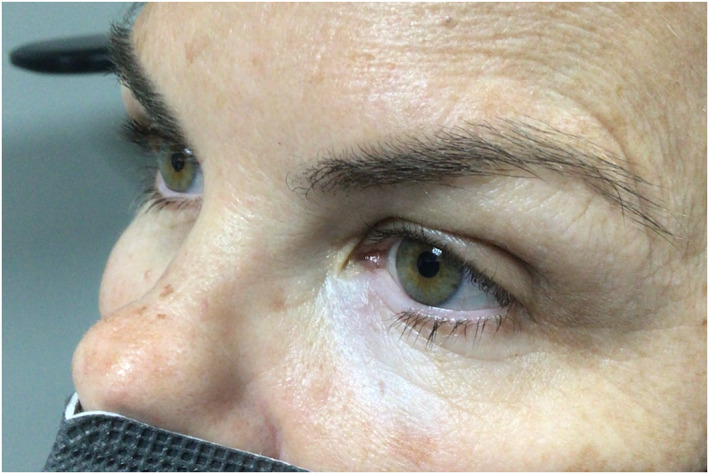

## 1 INTRODUCTION

Lichen sclerosus (LS), otherwise commonly called lichen sclerosus et atrophicus, is an inflammatory skin disease of unknown etiology described first in 1887 as a chronic, relapsing disease that predominantly affects the anogenital region.^1^ While lichen sclerosus is most often documented as a disease of the perineum, isolated extragenital lesions occur in approximately 6%–15% of patients, most commonly in locations such as the buttocks, thighs, neck, shoulder, upper torso, and wrists; facial involvement is rare.^1^, ^2^ The condition is more prevalent in women, and can occur at any age, with prepubertal children and postmenopausal women and men in their 40s being the most affected.^1^, ^2^, ^3^ The disease can cause skin atrophy, discoloration, scarring, functional impairment, and has the potential to become cancerous.^1^ Although the exact cause of LS is not yet fully understood, autoimmune factors may play a role in the development of the disease.^1^ A PubMed search of articles indexed for MEDLINE using the terms lichen, sclerosus, and eyelid and manually screened revealed 7 cases of lichen sclerosus involving the eyelid. We describe a case of lichen sclerosus of the lower eyelid involving the eyelash margin and its histopathology.

## 2 CASE REPORT

**FIGURE 1:**
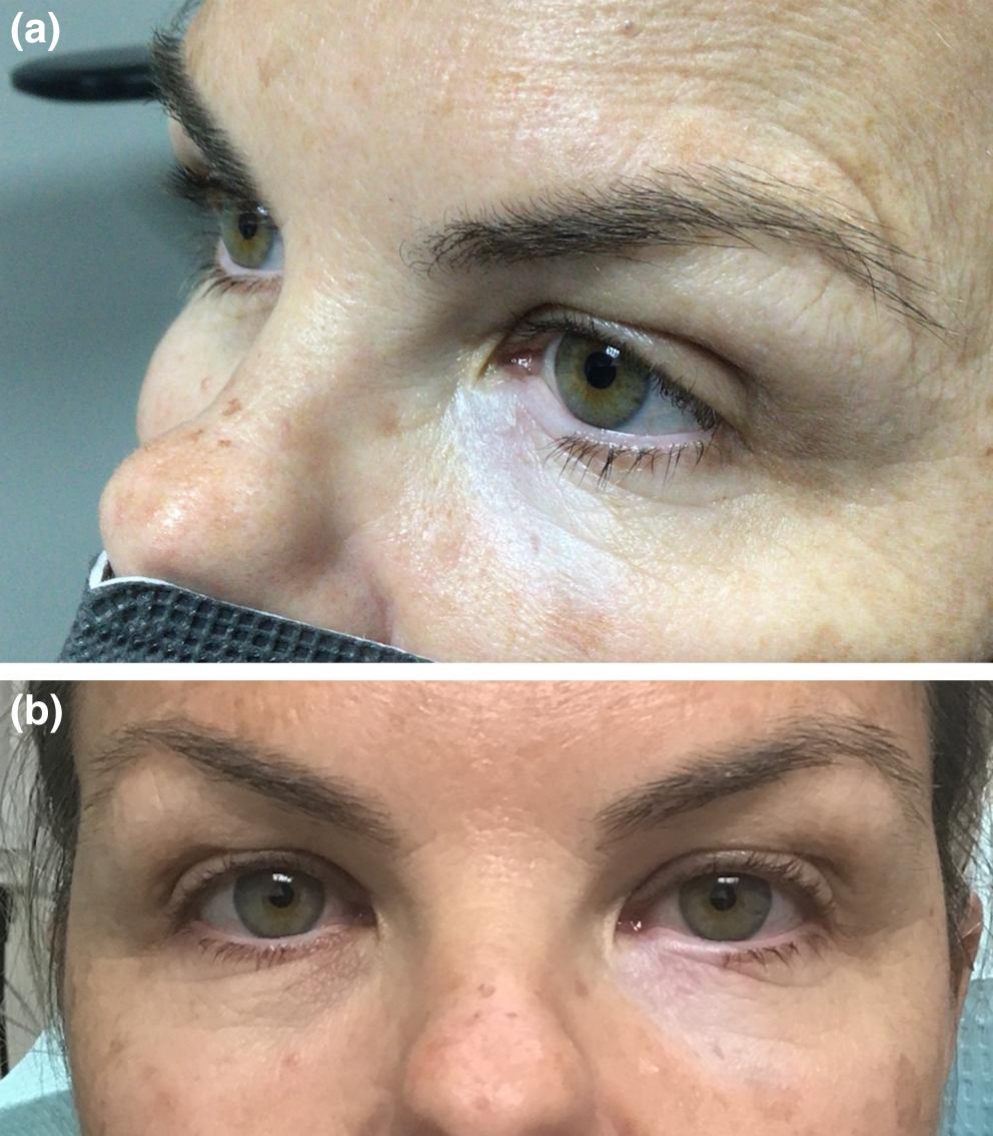
(a) Initial Ophthalmic examination. Flat, hypopigmented patch (32 × 20 mm) with scalloped borders involving the left lower eyelid and medial eyelash margin presenting in a 46‐year‐old patient. (b) Following initial punch biopsies, the left lower eyelid area remains without evidence of skin thickening, induration, or ulceration.

A 46‐year‐old female was self‐referred for evaluation of a left lower eyelid lesion of 8 months' duration. She first noted a small white patch under the eyelid involving the medial lower eyelash margin that had increased in size with associated loss of eyelashes. Her medical history included a family history of vitiligo (paternal aunt), but there was no history of ophthalmic conditions, autoimmune disease, trauma, or cancer. On ophthalmic examination there was a 32 × 20‐mm, flat, depigmented patch with scalloped borders involving the left lower eyelid margin that extended inferiorly. Madarosis of the medial lower eyelash margin was also noted. There was no evidence of skin thickening, induration, or ulceration (Figure 1). She underwent a punch biopsy, which was diagnosed as lichen sclerosus et atrophicus. Upon complete dermatological evaluation, no additional lesions were identified. Loteprednol 0.5% ophthalmic ointment was initiated. Low anticipation for disease progression, routine follow‐ups performed every 6–12 months during the patient's complete skin exams.

## 3 HISTOPATHOLOGY

**FIGURE 2:**
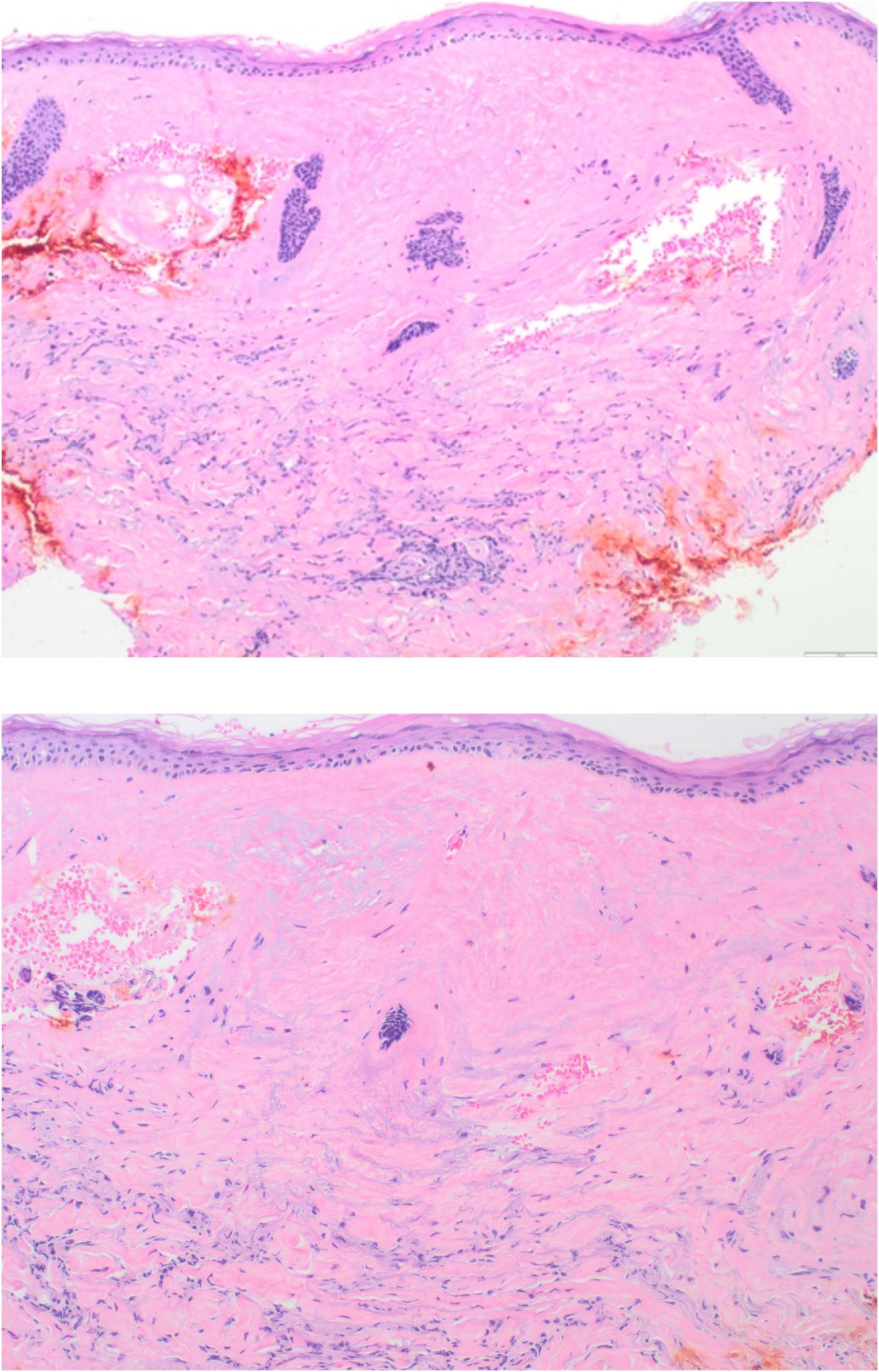
Initial biopsy. There is compact orthokeratosis and thinning of the epidermis. A broad zone of eosinophilic homogenized collagen is present in the superficial dermis with an underlying lymphoplasmacytic infiltrate.

Sections of the left lower eyelid lesion (Figure 2) demonstrated hyperkeratosis, thinning of the epidermis, and vacuolar alteration of the basal layer. Homogenization of the papillary dermis with an underlying lymphocytic infiltrate was present, consistent with lichen sclerosus. A well‐controlled Melan‐A immunohistochemical reaction labeled individual junctional melanocytes, excluding the diagnosis of vitiligo.

## 4 DISCUSSION

Lichen sclerosus is a debilitating and chronic disease that typically affects the anogenital area, although it can also be found on extragenital locations such as the shoulders, neck, trunk, breasts, and arms.^4^ Extragenital lichen sclerosus is typically asymptomatic with occasional pruritus, unlike anogenital lichen sclerosus which characteristically involve pruritus and dyspareunia.^1^, ^5^, ^6^ Autoimmune diseases such as vitiligo, alopecia areata, thyroid disease, and pernicious anemia are strongly associated with the pathogenesis of the disease, which supports a higher prevalence in females. However, genetic predisposition, trauma, chronic irritation, skin infections, and hormonal influences have also been associated with the development of lichen sclerosus.^1^, ^5^, ^6^ The extragenital lesions characteristically appear as well‐demarcated hypopigmented macules or plaques and are lesions thought to be prone to koebnerization, located in areas of physical trauma, pressure, and scarring.^1^, ^6^

Although facial involvement is rare, there have been a few reported cases of extragenital lichen sclerosus involving the infraorbital region in both men and women.^2^ Ophthalmic manifestations of lichen sclerosus have included lid notching, ectropion, acquired Brown syndrome, and associated keratoconjunctivitis sicca.^7^, ^8^, ^9^ To our knowledge, there are 7 documented cases of extragenital lichen sclerosus affecting the eyelid in medical literature.^2^, ^3^, ^6^, ^7^, ^10^, ^11^, ^12^ This is believed to be a novel and rare documented report of a patient with extragenital lichen sclerosus affecting the adnexa of the eye with eyelid and eyelash margin involvement.

The clinical differential diagnosis for hypopigmented patch also includes vitiligo, morphea (localized scleroderma), amelanotic melanoma, basal cell carcinoma, tinea versicolor, lichen planus, lichen simplex chronicus, and systemic scleroderma with eyelid involvement.^1^, ^7^ Vitiligo‐like lichen sclerosus (VLLS) is a variant of lichen sclerosus (LS) that presents with depigmented or hypopigmented patches resembling vitiligo. It is characterized by white, atrophic lesions that can occur in genital or extragenital areas. VLLS shares some clinical similarities with vitiligo, making it challenging to differentiate between the two conditions based solely on appearance. However, histopathological examination can reveal characteristic findings of lichen sclerosus, such as epidermal thinning, basal cell degeneration, and a lichenoid inflammatory infiltrate.^9^ It is important to distinguish lichen sclerosus from other conditions as some of them can have notable morbidity and/or mortality. For example, systemic sclerosus has the highest disease‐specific mortality among autoimmune connective tissue disorders.^13^ Meanwhile, morphea can lead to considerable morbidity, especially when it involves the head and neck, which increases the risk of neurologic and ocular complications including anterior uveitis.^13^ Moreover, to differentiate between LS and vitiligo, histopathological analysis using quantitative immunohistochemical techniques can be particularly useful. Lichen sclerosus typically shows alterations in the expression of certain proteins compared to vitiligo, such as increased expression of certain markers associated with inflammation and tissue damage, whereas vitiligo may show alterations in melanocyte‐related proteins.^9^ Clinically, LS often presents with white, atrophic, and sometimes wrinkled patches, commonly affecting genital or perianal regions, but also extragenital areas. Vitiligo, on the contrary, typically presents as depigmented macules or patches on various body surfaces, with a tendency to involve areas exposed to friction or trauma.^9^ LS may also present with symptoms of itching, pain, or discomfort, which are not typically associated with vitiligo.

Histopathology is useful to distinguish between these entities. Lichen sclerosus typically demonstrates epidermal atrophy, follicular plugging, homogenized collagen in the upper dermis with dermal edema, and lichenoid lymphocytic infiltrate.^1^ Lichen sclerosus and morphea overlap do not have distinct features and are typically classified based on their clinical presentation. Of importance, anogenital lichen sclerosus is associated with an increased risk of squamous cell carcinoma and verrucous carcinoma, but there have been no reported cases of malignant transformation of extragenital lichen sclerosus.^2^ Epidermal thickening, exaggerated basal atypia, and loss of the edematous‐hyaline layer are believed to be critical in the relationship between lichen sclerosus and carcinomas. While classic histologic features are seen in both vulvar and extragenital lesions, extragenital lichen sclerosus does not thicken to the same extent as the vulvar disease and is noted to have more epidermal atrophy, lower mitotic activity, less rete ridges, and decreased dermal hyalinization and fibrosis compared to the vulvar disease. Further dermoscopic and histopathologic studies indicate that the morphologic patterns of lichen sclerosus are influenced by location and duration of the disease.^14^

Initial treatment of lichen sclerosus involves topical corticosteroids for therapeutic control of the disease. If corticosteroid therapy is not effective or contraindicated, a topical calcineurin inhibitor like tacrolimus may be considered to avoid steroid‐induced side effects including hypopigmentation, glaucoma, undesirable skin atrophy.^2^ It is recommended that a collaborative approach between dermatology and internal medicine is taken to help identify any underlying systemic or multisystem issues.

## CONFLICT OF INTEREST STATEMENT

None to declare.

## AUTHOR CONTRIBUTIONS

**Tiffany Ho**: Data curation (lead); investigation (lead); methodology (lead); project administration (lead); resources (lead); visualization (lead); writing – original draft (lead); writing – review & editing (lead). **Susana Ortiz Urda**: Formal analysis (supporting); project administration (supporting); supervision (lead); validation (lead); writing – review & editing (supporting). **Ryan S. Berry**: Data curation (supporting); project administration (supporting); resources (supporting); supervision (supporting); validation (supporting); writing – review & editing (supporting).

## ETHICS STATEMENT

Not applicable.

## Data Availability

Data sharing not applicable to this article as no datasets were generated or analyzed during the current study.
